# Unidirectional Barbed Suture Versus Polyglactin 910 Suture for Vaginal Cuff Closure in Total Laparoscopic Hysterectomy

**DOI:** 10.7759/cureus.14257

**Published:** 2021-04-02

**Authors:** Kavita Khoiwal, Nirali Kapoor, Amrita Gaurav, Om Kumari, Jaya Chaturvedi

**Affiliations:** 1 Obstetrics and Gynaecology, All India Institute of Medical Sciences (AIIMS) Rishikesh, Rishikesh, IND

**Keywords:** vaginal cuff closure time, unidirectional barbed suture, v-loc suture, vicryl suture, operative time, total laparoscopic hysterectomy

## Abstract

Introduction and objective

Laparoscopic suturing of the vaginal cuff and knotting is the most challenging step in total laparoscopic hysterectomy (TLH) and requires surgical skill. The objective of this study was to compare the efficacy and safety of unidirectional barbed suture (V-Loc^TM^ 180; Covidien, Mansfield, MA) with the conventional polyglactin 910 suture (coated Vicryl; Covidien) for vaginal cuff closure in patients with benign uterine diseases undergoing total laparoscopic hysterectomy.

Methods

A prospective observational study was carried out at the department of obstetrics and gynecology, All India Institute of Medical Sciences (AIIMS), Rishikesh, for two years. A total of 109 patients with benign uterine diseases planned for TLH were included in the study. Laparoscopic vaginal cuff closure was performed with the unidirectional barbed suture (V-Loc) in 44 patients and with the standard polyglactin 910 suture (Vicryl) in 65 patients. The primary outcome measure was vaginal cuff closure time. Secondary outcome measures included total operative time, blood loss, average number of stitches, postoperative pain perception, duration of hospital stay, vaginal cuff-related complications, and dyspareunia.

Results

Demographic variables and baseline characteristics were similar in both groups except for body mass index (BMI). The mean vaginal cuff closure time was significantly less in the V-Loc group (8.84 ± 2.18 min) than in the Vicryl group (11.66 ± 1.74 min) (p = <0.01). Mean operative time was comparable in both groups (V-Loc group - 109.36±33.02 and Vicryl group - 108.49±40.48; p = 0.91). Other intraoperative parameters, such as blood loss and number of stitches in cuff closure, and postoperative characteristics, such as pain score, duration of hospital stay, vaginal cuff-related complications (vault cuff dehiscence, hematoma, or abscess), and dyspareunia, were comparable in both the groups.

Conclusions

The unidirectional barbed suture significantly reduces vaginal cuff closure time. It is a safe, effective, and well-tolerated alternative to conventional Vicryl suture for vaginal cuff closure in TLH without increasing the risk of postoperative vaginal complications particularly where affordability is not an issue and resources are accessible.

## Introduction

Hysterectomy is the most common gynecological surgery performed after cesarean section [1]. A minimally invasive approach is preferred, as it is associated with lesser complications, early recovery, less duration of hospital stay, and lower overall cost [2].

Laparoscopic suturing of the vaginal cuff and knotting is the most challenging step in total laparoscopic hysterectomy (TLH) and requires surgical skill. In TLH, the vaginal cuff can be sutured laparoscopically (either intracorporeal or extracorporeal) and vaginally. Suture material (e.g. polyglactin 910) and suturing technique (single/double layer or figure of eight) vary among surgeons and institutes. As vaginal cuff suturing constitutes a critical part of TLH, many techniques and variations in suture material have been developed to overcome surgical difficulties and associated complications.

Barbed sutures are the latest addition among sutures used for vaginal cuff closure. Conventionally, the Vicryl suture is used for this purpose in which knotting remains a major hurdle and needs expertise and a learning curve. Whereas barbed sutures are self-retaining, and knotting is not required due to its cutting barbs. Both bidirectional and unidirectional barbed sutures are available. Initially, the barbed suture was used in laparoscopic myomectomy [3] but its use in TLH for vaginal cuff closure is comparatively newer. Therefore, several studies have been published in the literature, which compares the utilization of barbed suture versus conventional Vicryl suture [4-15]. Most of these studies used bidirectional barbed suture, compared postoperative vaginal cuff complications after TLH, and determined that the barbed suture is a safe alternative to the Vicryl suture [4]. In this study, we compared the unidirectional barbed suture ( (V-Loc^TM^ 180; Covidien, Mansfield, MA) with the polyglactin 910 (coated Vicryl; Covidien) suture for laparoscopic vaginal cuff closure in TLH done for benign uterine diseases, in terms of vaginal cuff closure time and other intraoperative and post-operative outcome measures.

## Materials and methods

The study was a prospective observational study carried out at the department of obstetrics and gynecology, All India Institute of Medical Sciences (AIIMS), Rishikesh, Uttarakhand, for a duration of two years (May 2018 - April 2020). Prior approval was taken from the institutional ethical committee (AIIMS/IEC/18/548).

Patients with benign uterine pathology planned for TLH, who agreed to comply with the protocol, were able to communicate by telephone and answer questions, and were fit to withstand surgery were included in the study. While women with premalignant and malignant diseases of the uterus, cervix, or ovaries, a complex adnexal mass, pregnancy, genital prolapse, coagulation disorders, and who had any contraindication for laparoscopy were excluded. Informed and written consent was taken from all patients included in the study.

Patients were subjected to detailed history-taking, physical examination, blood investigations, pap smear, endometrial aspiration, imaging, and other standard preoperative workups. TLH was performed in all the patients by the same surgeons with a similar technique [16] except for the suture material used for vaginal vault closure. The vault was sutured laparoscopically in a single layer and in a continuous running manner with either unidirectional barbed suture (V-Loc^TM^ 180) or polyglactin 910 suture (coated Vicryl; Covidien). Hence, patients were categorized into two groups based on the suture material used (V-Loc group and Vicryl group). Both of the groups were then compared in terms of demographic variables and primary and secondary outcome measures. The primary outcome measure was vaginal cuff closure time (from the preparation of the needle for stitching to the cutting of suture at the end). Secondary outcome measures consisted of total operating time, blood loss, the average number of stitches used in vault closure, uterine weight, need for blood transfusion, conversion to laparotomy, hemoglobin difference, postoperative pain (VAS score), hospital stay, and complications such as pyrexia, urinary complaints, wound infection, vaginal discharge, vault dehiscence, hematoma, abscess, and dyspareunia. Patients were followed up after 10 days, six weeks, three months, and six months of surgery to assess postoperative parameters.

Statistical analysis

All calculations were performed using computer programs Microsoft Excel 2007 (Microsoft Corporation, Redmond, WA) and Statistical Package for the Social Science (SPSS) version 21.0 (IBM Corp., Armonk, NY).

Data were statistically described with respect to frequencies (number of cases) and percentages where appropriate. A chi-square test was applied to compare categorical data. When the expected frequency was <5, the Exact test was used instead. To compare quantitative variables, a t-test or Mann-Whitney test was used depending upon data distribution. A probability value (p-value) of less than 0.05 was believed to be statistically significant.

## Results

**Figure 1:**
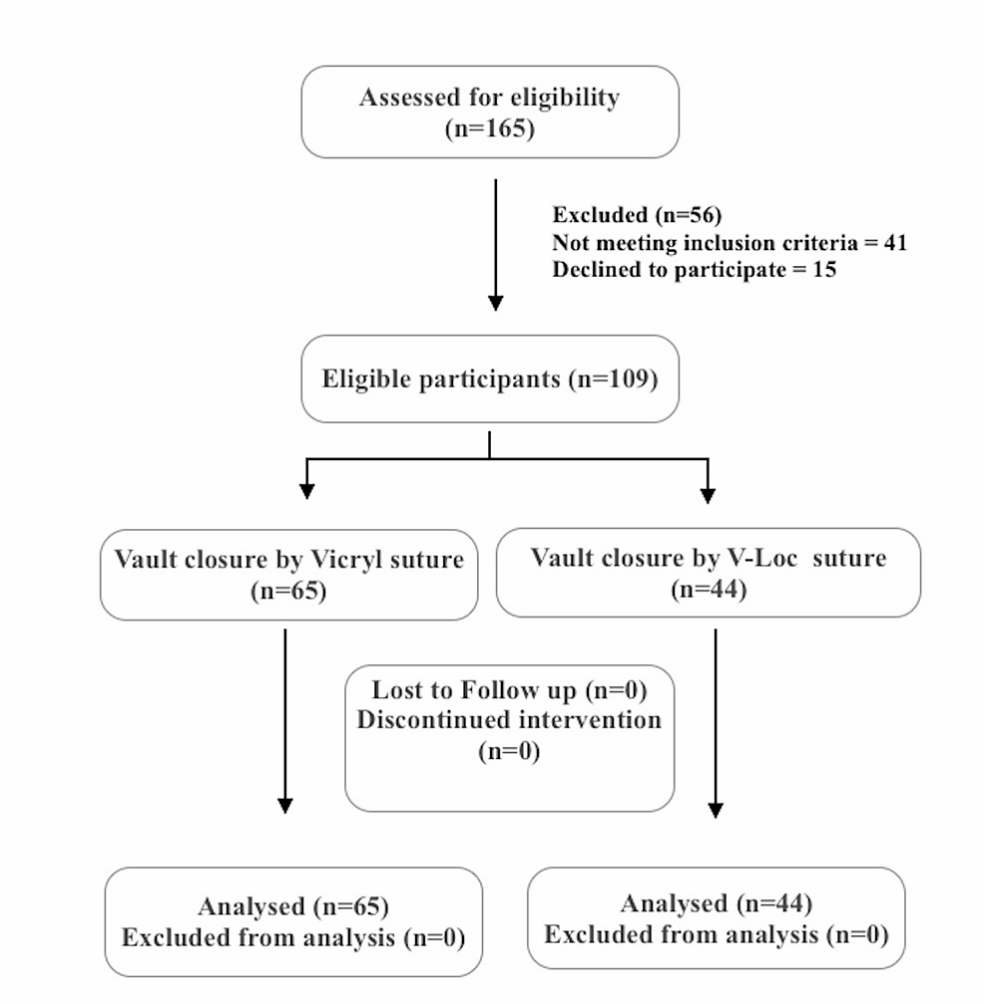
Flow chart of the study

A total of 165 women were evaluated for eligibility criteria, out of which 109 who fulfilled inclusion criteria were included in the study. Vaginal cuff (vault) closure was done with the V-Loc suture in 44 patients (V-Loc group) and with the Vicryl suture in the other 65 patients (Vicryl group). Figure 1 shows the flow chart of the study.

**Figure 2:**
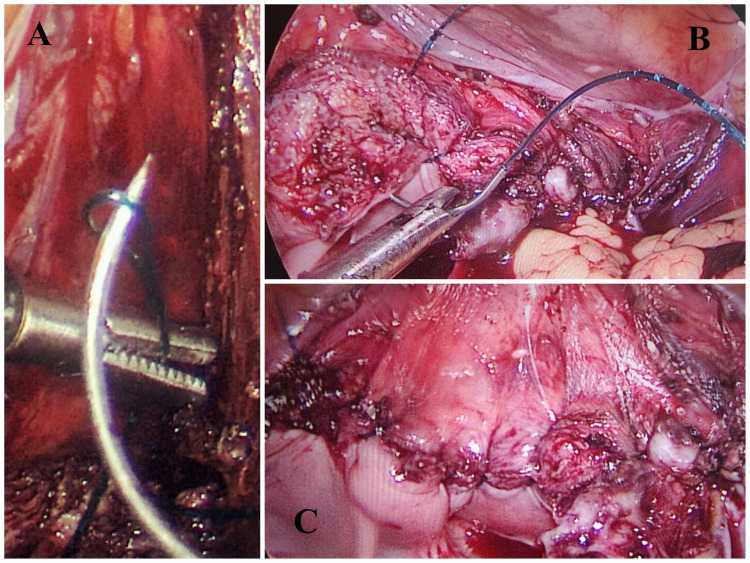
Operative image showing laparoscopic vaginal cuff closure using the unidirectional barbed suture (V-Loc)

**Figure 3:**
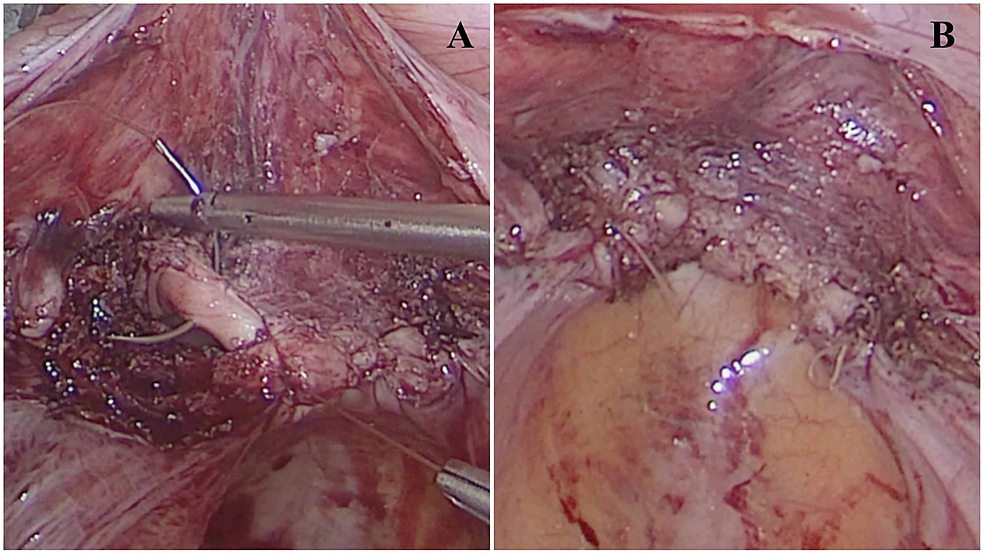
Operative image showing laparoscopic vaginal cuff closure using the polyglactin 910 (Vicryl) suture

Figures 2-3 show the operative images of the laparoscopic vaginal cuff closure using the V-Loc and Vicryl suture, respectively.

Demographic variables were comparable in both groups as shown in Table 1, except mean body mass index (BMI), which was significantly higher in the Vicryl group than the V-Loc group (p=0.02). However, it was not related to the suture preference, which might be a matter of chance.

**Table 1:** Demographic variables

Variables	Vicryl (n=65)	V-Loc (n=44)	p-value
Mean Age	45.39±7.2	43.86±7.15	0.28
Mean Body Mass Index	25.23±2.39	24.16±2.21	0.02
Primigravida	3 (4.6%)	0 (0.0%)	0.27
Multigravida	62 (95.4%)	44 (100%)	
Previous Cesarean	6 (9.2%)	4 (9.1%)	1.0
Past History, n (%)			
Hypertension	3 (4.6%)	7 (15.9%)	0.07
Diabetes Mellitus	4 (6.2%)	1(2.3%)	
Hypothyroidism	2 (3.1%)	6 (13.6%)	
Cholecystectomy	3 (4.6%)	2 (4.5%)	
Pelvic Surgery	0(0.0%)	2 (4.6%)	
Indication of surgery, n (%)			
Leiomyoma	50 (72.3%)	32 (68.2%)	0.84
Postmenopausal bleeding	3 (4.6%)	3 (6.8%)	
Adenomyosis	6 (9.2%)	5 (11.3%)	
Adnexal Masses	6 (9.2%)	4 (9.1%)	

Primary outcome measure

The vaginal cuff closure time was found to be significantly less in the V-Loc group (8.84 ± 2.18 min) in comparison to the vicryl group (11.66 ± 1.74 minutes) (p=<0.01) (Table 2).

**Table 2:** Operative variables

Variables	Vicryl (n=65)	V-Loc (n=44)	p-value
Mean vaginal cuff closure time (mins)	11.66±1.74	8.84±2.18	<0.01
Mean operative time (mins)	108.49±40.48	109.36±33.02	0.91
Mean blood loss (ml)	154.88±93.96	143.18±48.98	0.45
Average number of stitches	6.95± 0.69	7.23± 0.96	0.08
Conversion to laparotomy, n (%)	1 (1.5%)	0 (0.0%)	1.0
Blood transfusion, n (%)	6 (9.2%)	6 (13.6%)	0.54
Mean uterine weight (grams)	226.82±114.80	332.46±230.05	<0.01
Mean hemoglobin difference	1.23±1.37	1.31±0.72	0.74

Secondary outcome measures

Operative variables, such as overall operating time, blood loss, and hemoglobin difference, were comparable in both groups (Table 2) except uterine weight, which was significantly more in the V-Loc group than the Vicryl group (p-value <0.01). The choice of suture for vault closure was not based on the uterine weight; this difference might be a matter of chance.

In the present study, one patient in the Vicryl group required conversion to laparotomy due to uncontrolled intraoperative hemorrhage. However, laparotomy was done after vault closure to achieve hemostasis.

Postoperative variables, such as pain, hospital stay, and complications, were comparable in both groups (Table 3). At the six weeks, three months, and six months follow-up visits (physically or telephonically), no patients had vaginal discharge, vault hematoma, dehiscence, or abscess. No patient in either group had dyspareunia.

**Table 3:** Postoperative outcome variables VAS: visual analog scale

Variables	Vicryl (n=65)	V-Loc (n=44)	p-value
Mean VAS score	2.97±0.71	2.98±0.63	0.95
Mean hospital stay (days)	2.83±0.65	3.02±0.59	0.12
Complications at day 10			
Urinary complaints	4 (6.2%)	0	0.15
Wound infection	2 (3.1%)	0	0.51
Vaginal discharge	3 (4.6%)	1 (2.3%)	0.65

## Discussion

Barbed sutures are expensive yet considered safe and well-tolerated for vaginal cuff suturing [4], and they decrease operative time, as it overcomes a major hurdle of intracorporeal knotting with conventional sutures. However, passing the needle through the end of the loop of the unidirectional barbed suture (V-Loc) is also a necessary skill [17].

In the present study, we focused on the performance of the V-Loc suture on vault closure time primarily and found that V-Loc was easy to use and reduced vault closure time by 2.82 minutes than the Vicryl suture. The reduced time of suturing might be attributed to its knotless property and non-dependence of the surgeon on an assistant to hold the suture for tension. But it also depends on the surgeon’s expertise, as passing the needle through the loop of the V-Loc suture takes extra time at the beginning of the learning curve and comes with experience.

Laparoscopic vault closure time was significantly less in the V-Loc group than the Vicryl group in our study. Nevertheless, other intraoperative parameters like total operative time, blood loss, and the average number of stitches required for vault closure were not significantly different. Similar to our study, Park et al. also recorded significantly less vaginal cuff closure time with the V-Loc suture than the Vicryl suture (7.2 min vs 12.2 min; p = <0.001); closure time per stitch (0.5 min vs 1 min; P<0.001) [5]. They reported that surgery was significantly faster in the V-Loc group than the Vicryl group. Ardovino et al. compared the bidirectional barbed suture with the conventional suture using extracorporeal and intracorporeal knots and reported that the barbed suture takes 3.9 minutes on average for vaginal cuff closure in comparison to 6.2 minutes taken by conventional sutures (p = <0.001) [6]. Kim JH et al. also described a significantly lower vaginal cuff closure time with V-Loc (7.2±1.2 min) than the Vicryl suture (12.2±3.3 min) (p = <0.001), whereas the overall operative time was comparable in both groups (V-Loc - 91±50.3 min and Vicryl - 84.9±35.1 min; p = 0.354) [7]. The mean number of stitches was more with the V-Loc suture than the Vicryl suture (14.1 vs 12.3, p = <0.001) as it was in our study, though the difference was not significant.

In accordance with our study, Medina et al. did not find any significant difference in the overall surgical time (mean=181.8+51.7 min) in both suture groups [8]. Herraiz et al. also reported a similar overall mean surgery duration (112.08 min; range 60-240 min) in both the groups [9]. On the contrary, few studies reported a reduction in the total surgery time by using barbed sutures [4-5,10-12]. Smith et al. documented a decrease in total operating time by 15.6 minutes and vault closure time by 5.4 minutes using barbed sutures [10]. They suggested that this shortened surgical time may theoretically compensate for the elevated cost of barbed sutures. Zhou Y et al. also reported a shorter operative time with unidirectional barbed suture (220.2 vs 272.8 min) in a retrospective cohort study of 93 patients [11]. Similarly, Karacan T et al. found a significantly shorter duration of surgery in the V-Loc group than in the Vicryl group (p<0.05) irrespective of the experience of the surgeon [12]. Kim SM et al. also documented a significantly shorter operative time in the barbed suture group (p=0.002) in their retrospective study [4]. There are many factors and steps involved in TLH apart from the vault closure that may influence operation time, hence not many differences in total operative time were apparent in some studies including our own [7-9].

Intraoperative blood loss in our study was similar in both the groups, and it was in concurrence with the previous studies comparing V-Loc and Vicryl suture for vault closure [7-8].

The postoperative characteristics in our study, such as pain, were calculated by the visual analog scale (VAS) score, which was similar in both groups. Claudia CL et al. also reported similar pain perception in both groups [15]. The duration of hospital stay was also comparable in both groups, as reported by Kim JH et al. and Medina BC et al. [7-8].

Immediate postoperative complications, such as urinary infection, wound infection, and vaginal discharge, were comparatively more frequent in the Vicryl group than the V-Loc group (13.9% vs 2.3%) in our study but did not reach statistical significance. Claudia CL et al. also described a similar frequency of immediate postoperative complications in both groups [15].

In the present study, there were no cases of vault dehiscence, vault hematoma, vault abscess, and dyspareunia reported in both groups. Similarly, Kim JH et al. found no case of vaginal cuff dehiscence and a similar incidence of vaginal bleeding, cuff cellulitis, and postoperative fever in both groups in their study [7]. Bogliogo et al. [13] and Kim SM et al. [4] also found a similar rate of vaginal bleeding and vaginal cuff dehiscence in laparoscopic hysterectomy with or without the use of barbed sutures. Concurrently, Claudia CL et al. published similar incidences of vault dehiscence, cellulitis, hematoma, and abscess in both groups in a randomized controlled trial [15]. On the contrary, Martino M et al. documented a significantly less incidence of vaginal cuff dehiscence in the V-Loc group than in the Vicryl group (p=0.03) [14]. Karacan T et al. also recorded a higher rate of vaginal cuff dehiscence (3.3% vs 0) and infection or cellulitis (5.6% vs 0.9%) in the Vicryl group than the V-Loc group (p = <0.02) and concluded that the V-Loc suture is a safe, tolerable, and efficacious alternative to the conventional suture for vaginal cuff closure when used by residents/fellows as well as experienced surgeons [12]. Whereas Claudia CL et al. suggested no advantage of the barbed suture over the Vicryl suture in terms of operative time when performed by surgeons trained in intracorporeal laparoscopic suturing [15]. We postulate that both intracorporeal knotting with the vicryl suture and passing the needle through the loop of the V-Loc suture are vital skills and require a learning curve. Though the V-Loc suture decreases vault closure time, its use over the conventional Vicryl suture is based on the surgeon’s preference and availability of sutures.

The high cost is a potential drawback of the barbed suture, which stops its widespread use. However, the knotless property and non-dependence on the assistant make the barbed suture an easy, convenient, and safe substitute to the conventional vicryl suture.

Most of the studies reviewed were retrospective in nature [4,7,9,11-13]. Prospective nature was one of the strengths of our study. Moreover, we had an accurate study question (comparison of vault closure time). Pre-calculated sample size and randomization would have produced more robust results, which are probable shortcomings of our study.

## Conclusions

The unidirectional barbed suture significantly reduces vaginal cuff closure time. It is a safe, effective, and well-tolerated alternative to the conventional Vicryl suture for vaginal cuff closure in total laparoscopic hysterectomy without increasing the risk of postoperative vaginal complications, particularly where affordability is not an issue and resources are accessible.
